# Computer-Aided Approaches for Targeting HIVgp41

**DOI:** 10.3390/biology1020311

**Published:** 2012-08-20

**Authors:** William J. Allen, Robert C. Rizzo

**Affiliations:** 1 Department of Applied Mathematics and Statistics, Stony Brook University, Stony Brook, NY 11794, USA; Email: william.allen@stonybrook.edu; 2 Institute of Chemical Biology and Drug Discovery, Stony Brook University, Stony Brook, NY 11794, USA; 3 Laufer Center for Physical and Quantitative Biology, Stony Brook University, Stony Brook, NY 11794, USA

**Keywords:** HIV, AIDS, gp41, T20, structural biology, structure-based drug design, computer-aided drug design, molecular dynamics, docking, DOCK

## Abstract

Virus-cell fusion is the primary means by which the human immunodeficiency virus-1 (HIV) delivers its genetic material into the human T-cell host. Fusion is mediated in large part by the viral glycoprotein 41 (gp41) which advances through four distinct conformational states: (*i*) native, (*ii*) pre-hairpin intermediate, (*iii*) fusion active (fusogenic), and (*iv*) post-fusion. The pre-hairpin intermediate is a particularly attractive step for therapeutic intervention given that gp41 N-terminal heptad repeat (NHR) and C‑terminal heptad repeat (CHR) domains are transiently exposed prior to the formation of a six-helix bundle required for fusion. Most peptide-based inhibitors, including the FDA‑approved drug T20, target the intermediate and there are significant efforts to develop small molecule alternatives. Here, we review current approaches to studying interactions of inhibitors with gp41 with an emphasis on atomic-level computer modeling methods including molecular dynamics, free energy analysis, and docking. Atomistic modeling yields a unique level of structural and energetic detail, complementary to experimental approaches, which will be important for the design of improved next generation anti-HIV drugs.

## 1. Introduction

Infection with human immunodeficiency virus-1 (HIV), the causative agent of acquired immunodeficiency syndrome (AIDS) [1,2], is a significant global health threat. The World Health Organization (WHO) estimated that in 2010, 1.8 million deaths could be attributed to AIDS-related causes, and that approximately 34 million people worldwide were living with an HIV infection [3]. Nevertheless, the number of AIDS-related deaths has been on the decline since 2005 due to significant advances in antiretroviral therapies which were developed in large part using structure-based drug design [4]. The US Food and Drug Administration (FDA) recognizes six classes of antiretroviral drugs designed for the treatment of HIV infection: (*i*) nucleoside reverse transcriptase inhibitors (NRTIs), (*ii*) non-nucleoside reverse transcriptase inhibitors (NNRTIs), (*iii*) protease inhibitors (PIs), (*iv*) fusion inhibitors, (*v*) entry inhibitors, and (*vi*) integrase strand transfer inhibitors [5]. However, despite their successes, drug-resistant HIV mutants commonly arise during long-term clinical use of these therapies [6,7]. Moreover, many of these drugs are accompanied by adverse side effects, are expensive to produce, or, in the case of peptide fusion inhibitors, require injection to administer. Thus, the continued development of next-generation therapeutics is of paramount importance.

Fusion of the HIV outer envelope and the host cell membrane is an essential event for virus infection and proliferation. This process is driven by HIV glycoproteins 120 (gp120) and 41 (gp41). The sole FDA-approved member of the fusion inhibitor class of drugs, a 36-amino acid peptide called T20 (Fuzeon/Enfuvirtide) [8,9], selectively binds to gp41, blocking conformational changes required for virus-cell fusion [10,11]. Although T20 is effective in the short-term, problems inherent to peptide‑based drugs, and in particular drug resistance [12,13,14], have resulted in a significant effort to develop improved peptide as well as small-molecule fusion inhibitors. The development of next‑generation fusion inhibitors will rely heavily on a detailed understanding of gp41 structural biology. Computational modeling at the atomic level, taken in combination with experiment, can offer a unique and invaluable perspective on the structural biology of a protein drug target [15,16]. In fact, the design of T20 itself was motivated by an intimate knowledge of the conformational changes required for gp41 to mediate the fusion event [9].

There are many excellent reviews of HIV biology and in particular the fusion protein gp41; some notable recent examples include references [17,18,19,20,21,22]. The focus of this review is the structural biology of gp41 with special emphasis on the extracellular domain, and the link between available experimental models of the protein structure and atomistic computational techniques which exploit those models to aid in drug discovery.

## 2. Virus-Cell Fusion and Structural Biology of HIVgp41

### 2.1. HIV Envelope Proteins Originate from the env Gene

The HIV *env* gene is expressed in the host cell as a 160-kDa glycoprotein precursor (gp160), before it is proteolytically cleaved into two subunits by the human endoprotease furin [23]. The protein products—gp120 and gp41—self-assemble on the surface of the viral envelope as a trimer-of-heterodimers. Termed an envelope spike, the protein complex contains three membrane-spanning gp41 subunits interacting non-covalently with three extracellular gp120 subunits [24,25,26].

### 2.2. Virus-Cell Fusion is Initiated Through Receptor/Co-receptor Recognition

**Figure 1:**
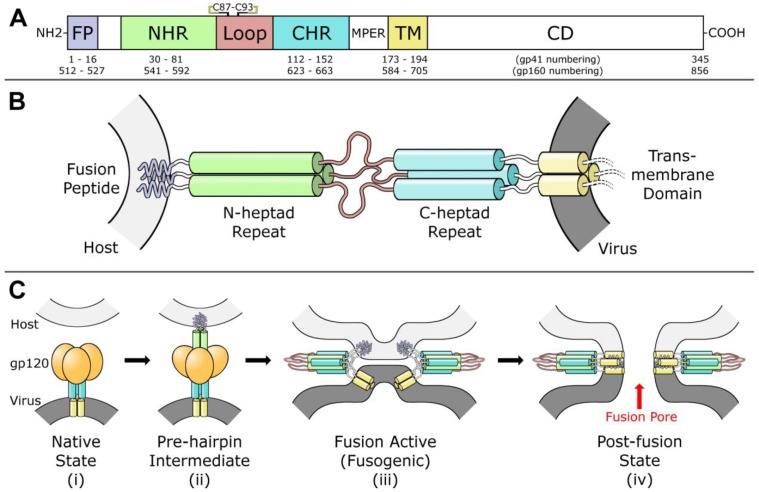
(**A**) Diagram of fusion protein gp41 sequence. From the N-terminus, the fusion peptide (FP), N-heptad repeat (NHR), loop region, C-heptad repeat (CHR), membrane-proximal external region (MPER), transmembrane domain (TM), and cytoplasmic domain (CD) are labeled. A disulfide bond between Cys 87 and Cys 93 in the loop region is indicated. (**B**) Model of gp41 trimer in the pre-hairpin intermediate conformation. In this model, gp41 spans from the host membrane (light gray) to the viral membrane (dark grey). Regions are colored according to the diagram in part (A). Cytoplasmic domain is omitted. (**C**) Model for gp41-mediated membrane fusion. In the native state (*i*) and the pre-hairpin intermediate (*ii*), gp120 receptors and co-receptors are omitted for clarity. In the fusion active state (*iii*) and the post-fusion state (*iv*), gp120 is omitted for clarity, and a second six-helix bundle is shown to illustrate cooperativity in forming the fusion pore. Red arrow indicates fusion pore. Concept for Figure adapted from Chan *et al*. [27].

Virus-cell fusion is mediated by gp41 and gp120 via advancement through four distinct conformational states: (*i*) native, (*ii*) pre-hairpin intermediate, (*iii*) fusion active (fusogenic), and (*iv*) post-fusion, as outlined in Figure 1C [27]. The gp120 first binds to primary receptor CD4 on the T‑cell surface, then undergoes a conformational change which exposes a chemokine co-receptor binding site specific to either CXCR4 or CCR5 [28,29]. Co-receptor binding initiates a cascade of major conformational changes in gp120 and gp41 [27,30,31]. Beginning from the native state in the envelope spike (Figure 1C-*i*), the fusion peptide (FP) and N-terminal heptad repeat (NHR) regions of the gp41 ectodomain (Figure 1A) extend outwards to form a trimeric helical bundle [32]. The FP initiates host cell membrane disruption either through oblique insertion [33] or by lateral insertion as an anti-parallel [34] or parallel β-sheet [35,36] in what is termed the pre-hairpin state (Figure 1B,C‑*ii*). This sequence of events is comparable to the ‘spring-loaded’ mechanism observed in other viral fusion proteins, including hemagglutinin [31,37]. Next, the three C-terminal heptad repeat (CHR) regions of the gp41 trimer, which form the stalk of the envelope spike in the native state, bind anti‑parallel in the grooves formed by the NHR trimeric bundle, thereby forming a six-helix bundle while simultaneously pulling the membranes into close proximity [38,39,40]. The formation of this bundle, also termed the fusion active conformation or fusogenic state (Figure 1C-*iii*), is highly stable and has been proposed as the rate limiting step of fusion [41]. Finally, the two outer membranes are fused in a two-part process [42,43,44] resulting in the post-fusion state. At this stage, there is a pore between the virus envelope and the host cell through which genetic material and other enzymes can pass (Figure 1C-*iv*). There is evidence that exactly one envelope spike is required to initiate pore formation [45], but other analyses suggest fusion is a concerted effort that requires anywhere from 2 to 19 envelope spikes [46,47,48,49]. Following budding from the T-cell host, the trimer-of-heterodimers are the only HIV proteins displayed on the virus outer envelope. HIV tropism (target cell recognition) is determined by gp120 alone [50]. It should be noted that there is a distinction in the literature between *gp160-numbering* and *gp41-numbering*. These designations refer to the residue index assigned to the first amino acid in the protein. In gp160-numbering, the first amino acid of gp41 is designated Ala 512; in gp41-numbering, the same amino acid is designated Ala 1. In this review, we use gp41-numbering derived specifically from the HIV-1 HXB2 isolate [51].

### 2.3. HIVgp41 as a Target for Fusion Inhibition

The extracellular regions of the envelope proteins are of pharmacological interest due to their accessibility to antibodies and other drugs. The gp41 pre-hairpin state is of particular interest because highly conserved regions in the NHR are transiently exposed at this stage [17,31,52]. It is at this step the fusion inhibitor T20 binds to the NHR, blocking formation of the six-helix bundle and transition into the fusogenic state [53]. It is important to note that T20 itself is identical in sequence to the gp41 CHR residues 127 to 162 [8,9]. Prompted by resistance observed with clinical treatment [14,54], next‑generation peptides were designed to include overlap with a highly conserved “deep pocket” on the surface of the gp41 NHR trimer [55] centered around residues *ca.* 54 to 70. Peptides including C34 and T1249 showed increased binding to T20-resistant mutants when compared to T20, but failed in clinical trials due to poor pharmacokinetic properties or adverse side effects [56,57]. However, the recently-developed peptide Sifuvirtide [58] which binds in the deep pocket has advanced to late clinical trials in China, and has shown promising anti-HIV activity against a variety of T20-resistant strains as well as low cytotoxicity [59,60].

In addition to peptide-based inhibitors, there is a major effort to design small molecule inhibitors of fusion [61,62,63,64,65,66,67,68,69,70,71]. Much of the focus has been in designing inhibitors that bind in the deep pocket [55]. Reportedly, small molecules which bind in that pocket sterically block formation of the six-helix bundle, thus disrupting fusion. However, it seems that none yet have high enough specificity or the appropriate drug-like properties to be used as effective therapeutics. In addition to peptides and small molecules, there is also a push toward development of covalent entrapment methods [72], small‑molecule/peptide chimeric molecules [73,74,75,76,77,78], as well as antibodies [79,80,81]. Specific examples of these fusion inhibitors and further discussions are extensively reviewed elsewhere [17].

## 3. Experimental Models of the gp41 Ectodomain

The foundation of structure-based drug design is a robust model of the system of interest—typically derived from experimental techniques such as x-ray crystallography, NMR, and electron microscopy. Since the discovery of HIV in 1983, many different constructs have been designed in an effort to solve the structure of gp41 and to study ligands binding to gp41. At the time of this writing (June, 2012), there are *ca.* 127 unique structures available on the Protein Data Bank (PDB [82,83]) containing HIVgp41 or gp41-derived peptides. With the exception of one NMR structure [65], complexes with small molecules have thus far proven elusive. At this time, no structure of the complete gp41 ectodomain is available. The structures and models that are available, however, provide valuable information for drug design as described below.

### 3.1. NHR/CHR Peptide Mixtures

In solution, peptides derived from the NHR alone will not preferentially trimerize. Instead, they tend to aggregate, impeding crystal formation [84]. However, when specific NHR-derived and CHR‑derived peptides are mixed in solution, they will form a six-helix bundle and, under the right conditions, grow crystals. The first gp41 six helix bundle structure was solved using this approach with peptides N36 (corresponding to gp41 NHR residues 35 to 70) and C34 (corresponding to CHR residues 117 to 150) [38]. Later, additional structures were solved of N36 in complex with certain C34 mutants [85,86] including Sifuvirtide [60], which was engineered with additional Arg and Glu residues to increase intra-helix salt bridge formation. Most recently, a novel six-helix bundle structure was obtained of T21 (corresponding to gp41 NHR residues 42 to 79) in complex with Cp621-652 (corresponding to gp41 CHR residues 110 to 141) [87]. These structures of the six-helix bundle have formed the foundation of our knowledge of the fusion-active and post-fusion conformations of gp41.

### 3.2. Fused NHR/CHR Constructs

**Figure 2:**
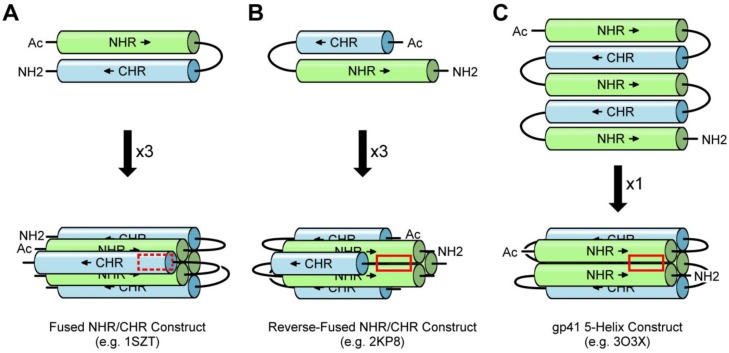
(**A**) In a fused NHR/CHR construct, the loop region is replaced by a short linker and the peptide trimerizes in solution, forming a six-helix bundle. In this structure, the conserved deep pocket is sterically blocked by a CHR peptide (dashed red box). (**B**) In the reverse-fused NHR/CHR construct, a truncated CHR peptide is linked N-terminal to the NHR. In solution, it will trimerize and the pocket is exposed (red box). (**C**) In the gp41 5‑helix construct, three NHR peptides and two CHR peptides are alternatively connected with short linkers. The construct folds into a five-helix bundle structure in solution leaving one NHR-groove and pocket exposed (red box). Arrows indicate peptide-bond direction from N-terminus to C-terminus.

NHR-derived and CHR-derived peptides, when fused by a short linker in place of the loop region, trimerize and fold into a six-helix bundle with increased thermostability over NHR/CHR peptide mixtures. This was first demonstrated with the construct N34(L6)C28 corresponding to NHR residues 35 to 68 fused by a short amino acid linker (SGGRGG) to CHR residues 117 to 144 [39,88,89,90,91,92,93]. This same construct was later expanded to include additional NHR and CHR residues, with or without the flexible linker, represented by constructs N36(L6)C34 [94], N45LC36 [93], gp41_528-683_ [95], and HR1‑54Q [96]. Each of these constructs, however, forms a structure in which the conserved deep pocket on the surface of the NHR trimer is blocked, potentially complicating small molecule screening efforts (Figure 2A). An alternative approach circumvents this problem by linking a truncated CHR‑derived peptide upstream from (in other words, N-terminal to) the NHR-derived peptide [65], thereby leaving the pocket exposed (Figure 2B). In yet another approach, three NHR-derived peptides (N36) and two CHR-derived peptides (C34) are alternatively connected by short amino acid linkers (either SGGRGG or GGKGGS) to create a five-helix bundle, leaving one deep pocket exposed [63,97,98,99] (Figure 2C).

### 3.3. Stabilized NHR Constructs

Wild type NHR-derived peptides only trimerize in the presence of, or when linked directly to, CHR-derived peptides. There are other methods, however, that stabilize NHR peptides to prevent aggregation and enable trimerization. The first approach is to fuse sequences from the gp41 NHR directly to a leucine zipper protein, GCN4, which is highly stable in solution [100]. An example of this is the construct pII41N which is comprised of 31 GCN4 residues fused N-terminal to gp41 NHR residues 30 to 79 [40,73]. A related construct, IQN17, consisting of 29 GCN4 residues fused N‑terminal to gp41 NHR residues 54 to 70, was designed to specifically display the conserved deep pocket in the trimeric structure for inhibitor development [101,102,103,104]. The second approach is through systematic mutation to either mimic the sequence of GCN4 [105], or increase overall helicity of the peptide by mutating numerous residues to alanine [106]. A third approach is by the rational engineering of the peptide backbone itself to include β-amino acids containing an extra carbon between the α-carbon and carbonyl-carbon [107]. Although these last two approaches are useful for understanding NHR trimerization or as potential inhibitors themselves, their utility as receptors in small-molecule drug discovery is likely limited due to low sequence conservation with active strains of HIV. Structures representing the core of the gp41 ectodomain (derived from NHR/CHR peptide mixtures, fused NHR/CHR constructs, and stabilized NHR constructs), including complete lists of PDB codes and accompanying citations, are summarized in Table 1.

**Table 1:** Summary of experimental HIVgp41 core structures available from the Protein Data Bank (PDB).

**NHR/CHR Peptide Mixtures**	
*Example Structure (PDB 1AIK):*	*Summary of PDB Structures:^1^*
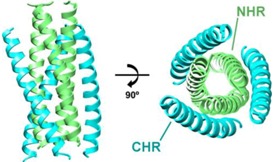	N36/C34: 1AIK [38].
	N36/C34-Mutants: 2ZZO [85 ]; 3AHA [86 ]; 2Z2T [N/A].
	N36/Sifuvirtide: 3VIE [60 ].
	T21/Cp621-652: 3VGX [87 ].
**Fused NHR/CHR Constructs**	
*Example Structure (PDB 1SZT):*	*Summary of PDB Structures:^1^*
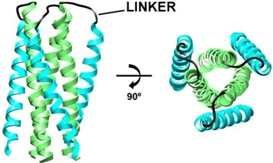	Standard Fused, e.g., N34(L6)C28: 1SZT [39]; 1QR8, 1QR9 [88]; 1DLB [89]; 1DF4, 1DF5 [90]; 1F23 [94]; 1I5X, 1I5Y [91]; 1K33, 1K34 [92]; 2OT5, 3CP1, 3CYO [93]; 2X7R [95]; 3K9A [96].
	Reverse Fused: 2KP8 (NMR) [65 ].
	5-Helix: 3O3X, 3O3Z, 3O40, 3O43 [98 ]; 4DZU, 4DZV [99 ]; 3O42 [N/A].
**Stabilized NHR Constructs**	
*Example Structure (PDB 1CE0):*	*Summary of PDB Structures:^1^*
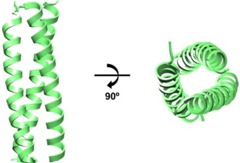	Chimeras with GCN4 (pII41N): 1ENV [40]; 1FAV [73].
	Chimeras with GCN4 (IQN17): 1CZQ, 2Q3I; 2Q5U, 2Q7C [101 ]; 1GZL [102 ]; 2R3C, 2R5B, 2R5D [103 ]; 3L35, 3L36, 3L37 [104 ].
	Heavily Mutated: 1CE0 [105 ]; 2ZFC [106 ].
	α/β-Peptide Foldamers: 3F4Y, 3F4Z, 3F50, 3G7A [107 ]; 3O3Y [N/A].

^1^ Structures were solved by X-ray diffraction unless otherwise noted. NMR: Nuclear magnetic resonance. N/A: Structure available on PDB without accompanying citation.

### 3.4. Antibody-Bound gp41-Derived Peptides

Several groups have reported structures of fragment antigen-binding (Fab) regions from antibodies in complex with short peptides derived from the gp41 MPER region, including Fabs 2F5 [81,108,109,110,111], 4e10 [79,112], 13H11, and Z13e1 [80]. Additionally, several structures have been reported of a Fab bound to a gp41 multimeric helix bundle representing the core of the ectodomain [113,114,115,116]. Although their application to design of small molecule or peptide inhibitors has yet to be fully exploited, these structures will be important for the future development of improved antibodies or, possibly, an HIV vaccine.

### 3.5. Apo gp41-Derived Peptides

Another class of experimental structures involves short, unliganded (apo) gp41-derived peptides which do not form a biologically-relevant trimer or hexamer. Although their direct application in small molecule drug design has been limited, they contribute to the understanding of the gp41 fusion event, which in turn can be useful for future inhibitor design. For example, it was observed that the gp41 FP conformation is likely helical when adsorbed into an SDS micelle representing the host cell bilayer [117]. In addition, part of the gp41 MPER prefers to remain helical in solution, but does not have a strong propensity to self-associate [118]. This enables flexibility in the MPER and C-terminal end of the CHR region, which is likely a key characteristic of the six-helix bundle formation mechanism. Other peptide structures from the FP [119,120,121,122], loop region [123], MPER [124,125,126,127], and CTD [128] have also been reported. A complete list of these apo gp41-derived peptides, as well as the antibody-bound gp41-derived peptides including complete lists of PDB codes and accompanying citations, are summarized in Table 2.

**Table 2:** Summary of antibody-bound peptides and apo-peptides derived from HIVgp41 available from the PDB.

**Antibody-bound gp41-derived Peptides**			
*Antibody:*	*Target:*		*Summary of PDB Codes:^1^*
2F5	MPER		1TJG, 1TJH, 1TJI [108]; 2P8L, 2P8M, 2P8P, 3D0L, 3D0V, 3DRO, 3DRQ [109]; 1U8H, 1U8I, 1U8J, 1U8L, 1U8M, 1U8N, 1U8O, 1U8P, 1U8Q, 1U91, 1U92, 1U93, 1U95, 2F5B, 2PW1, 2PW2, 3IDG, 3IDI, 3IDJ, 3IDM, 3IDN [110]; 3DRT, 3EGS [111]; 3LEX, 3LEY [81]; 1U8K, 3MOA, 3MOB, 3MOD [N/A].
4e10	MPER		1TZG [112]; 2FX7, 2FX8, 2FX9 [79].
13H11	MPER		3MNW, 3MNZ, 3MO1 [N/A].
Z13e1	MPER		3FN0 [80].
Various	gp41 multimer		2CMR [113]; 2XRA [114]; 3MA9, 3MAC [115]; 3P30 [116].
**Apo gp41-derived Peptides**			
*Peptide Origin:*		*Summary of PDB Codes:^1^*	
FP		1ERF (IR) [119]; 1P5A (IR) [120]; 2ARI (NMR) [117]; 2PJV (NMR) [121]; 2JNR (NMR) [122].	
Loop		1IM7 (NMR), 1J8N (NMR), 1J8Z (NMR), 1J9V (NMR), 1JAA (NMR), 1JAR (NMR), 1JC8 (NMR), 1JCP (NMR), 1JD8 (NMR), 1JDK (NMR) [123].	
MPER		1JAU (NMR), 1JAV (NMR) [124]; 1LB0 (NMR), 1LCX (NMR) [118]; 1MZI (NMR) [125]; 2PV6 (NMR) [126]; 3G9R [127].	
CTD		3GWO, 3H00, 3H01 [128].	

^1^ Structures were solved by X-ray diffraction unless otherwise noted. IR: Infrared spectroscopy; NMR: nuclear magnetic resonance. N/A: Structure available on PDB without accompanying citation.

### 3.6. Electron Microscopy-Derived Models

Cryo-electron microscopy (cryo-EM) represents another powerful experimental approach which, in particular, has provided key structural and stoichiometric details for the HIV envelope spike formed by gp41 and gp120 [24,25,26,129,130]. Importantly, groups have used cryo-EM models in conjunction with x-ray crystallographic structures to investigate the gp120 oligomerization state [131] and specific loop conformations [132]. In addition to HIV, some experimentalists have also studied the simian immunodeficiency virus (SIV) as a model system due to its high sequence identity and its relative abundance of envelope spikes on the viral surface (73 ± 25 for SIV compared to 14 ± 7 for HIV) [25]. The base of the envelope spike structure (most proximal to the viral membrane), which is formed by a trimer of gp41 MPERs, has been reported both as a single compact stalk in which the MPERs are closely associated [24,130], or as a tripod-like configuration in which they are more open [25,26]. Some researchers have suggested [26] that the compact stalk structures may have bias as a result of the specific reference employed and symmetry enforcement methods used in the refinement. On the other hand, potential issues with the tripod-like configuration have been noted [133] as a result of limitations of the experimental data collection and post-processing strategies used to construct the model. Although further experiments will be required to resolve these discrepancies, cryo-EM provides an important and unique insight into the structure of the HIV envelope spike which can facilitate the design of antibodies and other therapeutics.

## 4. Computational Modeling of gp41 and Fusion Inhibitors

Computational modeling makes use of structural models in combination with physico-chemical properties and computer algorithms to make predictions of molecular interactions. Discussions below focus on a select subset of the many studies of gp41 and fusion inhibitors, with an emphasis on those describing molecular recognition in the context of drug design.

### 4.1. Interactions of Small Molecule Inhibitors with gp41

**Figure 3:**
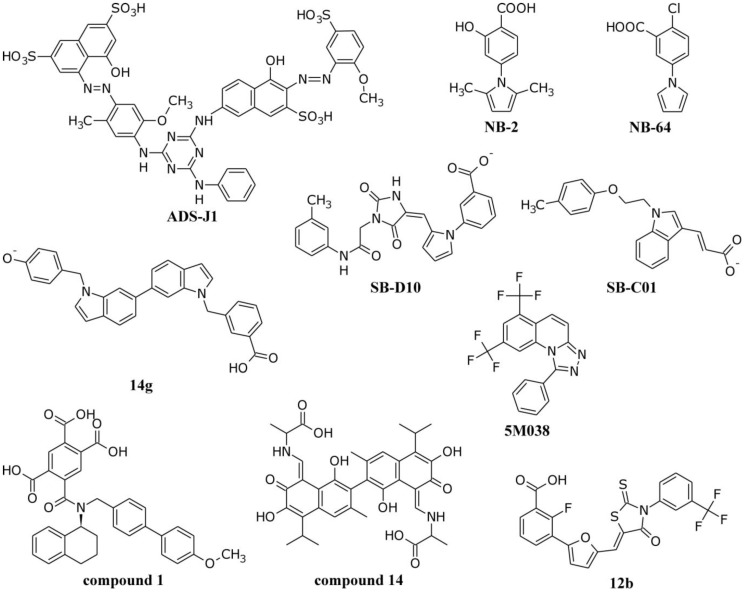
Representative chemical structures with reported gp41-binding activity. (ADS-J1 [61]; NB-2, NB-64 [62]; SB-D10, SB-C01 [69]; 14g [153]; 5M038 [63]; compound 1 [65]; compound 14 [71]; 12b [67]).

In the first published virtual screen to the conserved deep pocket on gp41, Debnath, Jiang and coworkers [61,134] screened a library of 20,000 small organic compounds using the virtual screening program DOCK3.5 [135,136]. The receptor model used in this study was 1AIK [38] with one CHR‑derived peptide removed. The 200 best compounds as determined by a non-bonded molecular mechanics scoring function were visually inspected, and sixteen were purchased for experimental testing. One of the compounds, ADS-J1 (Figure 3), exhibited encouraging cytotoxicity and IC_50_ profiles, but as the original authors note, its high molecular weight (1,177 Da) prevented it from becoming a drug lead, although it is still widely used as a control compound for evaluating six-helix bundle formation [17]. A later study suggested that an alternative mechanism of fusion inhibition adopted by ADS-J1 was not through binding the pocket on gp41, but instead through binding to the V3 loop of gp120, thereby disrupting interactions with the co-receptor [137]. However it was ultimately confirmed through a combination of experiment and docking with the program Glide [138,139] that ADS-J1 does in fact bind in the conserved pocket region on the NHR trimer and that it prevents cell fusion by obstructing six-helix bundle formation [140].

Another key study reported by Jiang *et al.* [62] used a high-throughput assay to identify several N‑substituted pyrrole derivatives as candidates to disrupt six-helix bundle formation. Top compounds (including NB-2 and NB-64; see Figure 3) were then docked into the deep pocket of 1AIK using the program Glide [138,139] to identify the most likely binding poses. Importantly, the predicted binding poses of both NB-2 and NB-64 included a salt bridge between the acidic groups of the small molecules and Lys 63. In the native state, Lys 63 forms a highly conserved salt bridge with Asp 121 from the CHR region—an interaction that is essential for gp41-mediated fusion [141]. This finding demonstrated the utility of considering native CHR-residue interactions in the pocket during drug discovery. However, two later studies each proposed that NB-2 adopts a different binding pose wherein the acidic group forms a salt bridge with Arg 68 [142,143]. In fact, one group used that alternative orientation in a 3D-QSAR model to establish a quantitative correlation with experiment (*R*^2^ = 0.984) for a series of congeneric inhibitors based off of NB-2 [143]. Thus, uncertainties remain in the correct binding pose of N-substituted pyrrole derivatives.

The largest published virtual screen to the gp41 deep pocket to date was recently performed by Holden *et al.* [69]. They screened *ca.* 500,000 compounds from the ZINC database [144] using DOCK6 [145,146] and the receptor model 1AIK (with one CHR peptide removed). Unlike a traditional virtual screen, however, the authors re-scored and re-ranked the results based on not only the sum of all interactions in the binding site, but on how similar (in identity and magnitude) those interactions were to the interactions formed by native CHR residues Trp 117, Trp 120, Asp 121, and Ile 124. This procedure stemmed from the idea that it is not only important to identify molecules which interact strongly in a binding site, it is also important to identify molecules which interact *in a specific manner* in a binding site—especially forming contacts with the most conserved residues. After purchasing 115 compounds, 7 leads were identified with promising cytotoxicity, cell-cell fusion, and activity profiles [69]. Interestingly, one of the compounds (denoted SB-D10) contained an N‑substituted pyrrole scaffold which was remarkably similar in structure to NB-64 (Figure 3). The docked pose of SB-D10 predicted that the acid group formed a salt bridge with Lys 63, much like that which was originally proposed for NB-64 [62]. Another compound from the same study with high geometric overlap between its acidic group and the position of Asp 121 is compound SB-C01. This and other representative small molecules with reported anti-fusion activity including 5M038 (Frey *et al.* [63]), compound 1 (Stewart *et al.* [65]), compound 14 (Yang *et al.* [71]), and 12b (Jiang *et al.* [67]) are shown in Figure 3.

Expanding on traditional docking calculations, Tan *et al.* [147] first docked a series of four ADS-J1 analogs to the gp41 deep pocket using the program AutoDock [148] and the receptor model 1GZL [102] (an IQN17 construct), then performed energy minimization and molecular dynamics simulation followed by MM-PBSA [149] free energy analysis. From these calculations, the authors were able to identify polar and nonpolar contributions from specific residues which were most important for determining inhibitor specificity. For example, they identified Ile 62 as a major determinant of ligand binding through forming hydrophobic interactions. Previously, it was demonstrated experimentally that when Ile 62 is mutated to a hydrophilic residue, the capacity for gp41 to form a six-helix bundle is greatly reduced [150]. Other similar computational efforts identified the importance of Gln 64 and Gln 66 in forming hydrogen bonds with certain inhibitors [151], and the importance of Trp 60 in forming a hydrophobic contact with other inhibitors [152]. Taken together, these and similar studies can help guide the development of future drug leads.

In an orthogonal approach, Tan *et al.* [154] used *de novo* design in an attempt to create new analogues of the N-substituted pyrrole inhibitor with increased binding affinities to the gp41 deep pocket. Although the compounds that they ultimately synthesized and tested presented less activity than the original NB-2, *de novo* approaches are attractive in that unique series of compounds can be developed that may not have otherwise been identified in a virtual screen.

While most groups have used 1AIK as the receptor model to identify likely binding poses for small molecules [64,67,155,156,157], other constructs have also been used. For example, Zhou *et al.* [153] docked a series of congeneric indole-based compounds (including 14g, Figure 3) to two different models of gp41 including 2R5D (an IQN17 construct) and 3P7K [158]. Here, the authors reported that they rotated the side chain of Lys 63 prior to docking such that it would interact more favorably with their small molecules in the binding site. In another study, Gochin *et al.* [159] reported docking to three different constructs (2R53, 3P7K, and 2KP8) in order to generate an ensemble of results and improve sampling. An important consideration in docking or virtual screening experiments is the appropriate selection of an experimental structure to model the receptor, as use of different receptors will likely influence the final results. When overlaid, models of the gp41 ectodomain core may contain alternative side chain conformations, including in the deep pocket, especially when models originate from different types of experimental constructs. Therefore, in the absence of a fully-flexibly receptor model during docking or virtual screening, it may be most appropriate to use a structure of a bound NHR trimer with the ligand removed (e.g., 1AIK with a CHR peptide removed).

**Figure 4:**
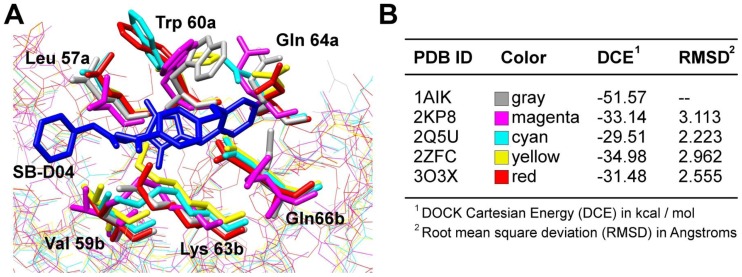
(**A**) Docked pose of compound SB-D04 (blue) [69] in PDB structure 1AIK (gray). Structures 2KP8 (magenta), 2Q5U (cyan), 2ZFC (yellow), and 3O3X (red) are overlaid. (**B**) DOCK energies and RMSDs associated with energy-minimized SB-D04 in complex with different PDB structures.

To illustrate this point, we aligned five gp41 structures from the PDB (1AIK, 2KP8, 2Q5U, 2ZFC, and 3O3X) along the backbone α-carbons. The structure 1AIK contains CHR-derived peptides, and the structure 2KP8 contains an NMR model of a small molecule bound, both of which were removed. The structures 2Q5U, 2ZFC, and 3O3X all contain deep pockets which are unliganded and solvent-exposed. A small molecule inhibitor (SB-D04) with known activity [69] which was previously docked into 1AIK was overlaid with the binding sites of the other four gp41 models (Figure 4A). Each pose was energy minimized and a non-bonded molecular-mechanics energy was computed between the ligand and the different gp41 constructs using DOCK6 [145,146]. Without accounting for flexible side chains, different crystal models of the receptor resulted in significantly different docked energies and the minimized binding geometries showed greater than expected movement (>2 Å, Figure 4B). In general, a potential concern is that use of different receptor models in a virtual screen could lead to identification of different compounds for purchase and experimental testing. Several docking programs including AutoDock [148] and Glide [138,139] account for receptor flexibility by allowing certain side chain torsions to move. Efforts to effectively include multiple receptor conformations into DOCK are ongoing.

### 4.2. Molecular Dynamics Simulations of the gp41 NHR/CHR Core

Molecular dynamics (MD) simulations of the gp41 ectodomain core (e.g., the NHR trimer or six‑helix bundle) are important for quantifying binding modes of peptide inhibitors, elucidating origins of affinity, or used to probe mechanisms of resistance. In an early study, Siebert *et al.* [160] performed MD simulations of a short segment of the NHR trimer derived from PDB structure 1CZQ (an IQN17 construct). Following simulation, the authors post-processed the ensemble of snapshots with the test particle insertion method [161] to identify regions in and around the deep pocket with the highest hydrophobicity. Several native CHR residues which are known to be important to six-helix bundle formation (including Trp 117, Trp 120, and Ile 124) bind directly in the predicted hydrophobic sites. Additionally, the authors identified a site immediately adjacent to the deep pocket with high hydrophobic character which is occupied by Tyr 127 of the native CHR. Extending inhibitors from the deep pocket into this site may be a promising method for improving currently available small molecule leads.

Experimentally, Chan *et al.* [55] determined differential viral entry activities for the peptide inhibitor C34 (corresponding to CHR residues 117 to 150) and five C34 mutants (W120F, W120L, W120V, W120A, and W120G). To identify the origins of the differential binding affinities, Strockbine *et al.* [162] performed MD simulations of the NHR trimer in complex with either wild type C34 or one of the above five C34 mutants, followed by MM-GBSA [149] free energy calculations. Through a residue-by-residue decomposition of interaction energies, the authors determined that mutations at position Trp120 only affected local hydrophobic interactions within the deep pocket of the NHR trimer, and that the effects of the mutations were not propagated to other regions of the peptide. Thus, this experiment illustrates the importance of contacts made at that specific position in future inhibitor design. Following this example, Watabe *et al.* [85] experimentally determined that C34 mutant S138A had approximately four-fold greater activity over wild type C34 against a T20-resistant strain of HIV. After reporting the crystal structure for C34 S138A in complex with N36 (2ZZO), the authors performed a single point energy calculation to decompose specific interactions between the mutated peptide and NHR residues. When compared to the same energy decomposition for wild type C34 (from 1AIK), they determined that despite a small loss in electrostatic energy between C34 S138A and NHR residue Glu 49, a large gain in van der Waals interaction energy between C34 S138A and NHR residue Leu 45 drove the differential binding affinities. Another study also demonstrated that interactions in the conserved pocket could be modulated through rational crosslinking of a short CHR peptide [163], overall improving binding affinity.

Several computational studies have sought to explain the binding mode and key interactions between the FDA‑approved inhibitor T20 (which does not interact in the deep pocket) and gp41. McGillick *et al.* [164], building on early key work of Caffrey *et al.* [165,166,167], reported the first atomistic complex of T20 with gp41, which was embedded in an explicit lipid bilayer, and successfully used MD simulations to delineate which structural and energetic features lead to resistance for seven deleterious point mutations (L33Q, L33S, G36V, I37K, V38E, Q40H, and Q40K). Prior to this study, mechanisms of resistance to T20 were not well understood because a bound complex with gp41 was unavailable. A later experimental structure [95] consisting of a CHR peptide containing all T20 residues in complex with the NHR region of gp41 validated the computational model. Qiu *et al.* [168] subsequently studied the same seven mutations and demonstrated that in the case of T20 binding, mutants I37K and Q41R were the greatest contributors to loss of interaction energy. Experimentally-observed mutations V38E and N43D introduced electrostatic repulsions between the NHR receptor and T20, reducing binding affinity. Interestingly, the authors of this latter study also observed that the C-terminal 8 residues (WASLWNWF) of T20 become uncoiled when bound to the NHR trimer. This observation, however, could be a result of simulations not including a explicit lipid bilayer. The earlier study by McGillick noted significant favorable interactions between T20 and lipids which likely stabilize the overall complex.

### 4.3. Molecular Dynamics Simulations of the gp41 Fusion Peptide

Although there have been numerous studies, there is no clear experimental consensus on the secondary or tertiary structure of the gp41 FP. Short fragments (23 to 30 residues) from the N-terminus of gp41 freely adopt α-helical conformations [117,119,121], β-strand conformations [120,169], or combinations of both [170] depending on the experimental conditions and oligomeric state. There is debate as well over whether a parallel or anti-parallel β-sheet conformation [34,35,36], an α-helical conformation [33,121], or a uncoiled conformation [171] is fusogenic. There is further evidence to suggest that the FP structures observed experimentally thus far are contingent on the model peptide length; and that perhaps the insertion depth rather than structure is a greater determinant for fusogenicity [172]. The question of FP secondary structure is complicated even further by the fact that studies are typically not performed in the context of the biologically-relevant trimer. MD simulations are particularly well-poised to investigate the structure of the FP and the mechanism of fusion at an atomic level under a variety of conditions.

Kamath and Wong [173,174] performed MD simulations of the gp41 FP (residues 1 to 16) to determine its interaction with a lipid bilayer. They predicted that an oblique insertion beginning from the α-helical conformation is the most likely mechanism of fusion. In addition, there are many parallels between the mechanism they propose and the mechanism of hemagglutinin membrane fusion/fusion peptide insertion, which also occurs obliquely [175]. In fact the secondary structure of the gp41 FP proposed in this study is strikingly similar to the secondary structure of the influenza virus hemagglutinin amino acids of the same region [173]. Once inserted into the bilayer, there is evidence that conformational flexibility, rather than secondary structure, is more important to pore formation [174]. The glycine and alanine richness of the FP (residues 1 to 16) contributes to this conformational flexibility. They also find that the secondary structure does not change significantly between wild type fusion peptide and two inactive mutant fusion peptides, suggesting that secondary structure is not the primary driving force for virus-cell fusion. Rather, the angle of insertion and the effect mitigated on the bilayer—a thinning through interaction with the lipid hydrophobic tails—determines fusogenicity [173].

More recently, Grasnick *et al.* [176] used MD simulations and NMR experiments to demonstrate that the gp41 FP structure is a combination of random coil and true α-helix. Their study indicates that the FP prefers, when inserted into a bilayer, an irregular coiled form. However, their system, and the system presented by Kamath and Wong [173,174], each only contains one model FP. Thus, these models do not account for interactions between FPs including the potential formation of tertiary structures and other supra-molecular oligomeric states.

In another study, Venken *et al.* [177] used MM-PBSA [149] free energy analysis to quantify the energy of interaction between VIRIP, a naturally occurring antiretroviral peptide [122], and the gp41 FP. They were able to successfully replicate binding trends computationally from experimental data. Further, they were able to use their model to predict, and later experimentally verify, mutated forms of VIRIP that would bind more strongly to gp41. This approach could be valuable to the future development of peptide-based inhibitors targeting the FP.

### 4.4. Molecular Dynamics Simulations of the Transmembrane Domain

Kim *et al.* [178] performed MD simulations and experiment to study the self-association behavior of the gp41 TM domain (residues 174 to 195) in a lipid bilayer. They found that a trimeric bundle is likely the most stable configuration for the TM domain in the bilayer. Trimerization is facilitated by inter-chain hydrogen bonds between conserved arginine residues (Arg 185) that only occur in the right-handed helical bundle. The self-association and stability of this bundle could help gp41 trimerize in the HIV envelope.

### 4.5. Molecular Dynamics Simulations of T20 and Other CHR-Derived Peptides

In addition to simulations of gp41, simulations of T20 and various other peptide-based fusion inhibitors have been performed to either understand their interaction with the membrane or optimize behavior. Conceptually, a CHR-derived peptide with higher α-helix propensity would bind more readily to the gp41 NHR trimer because it pays less of an entropic cost before binding. Martins do Canto *et al.* [179,180,181] performed MD simulations of T20 and T1249 alone in solution and in the presence of a model lipid bilayer. In solution they found that the peptides were both mostly disordered, with sporadic formation of turn and bend configurations. In the presence of the membrane, however, both peptides overwhelmingly preferred to adopt an amphipathic π-helix configuration. To improve helicity, Singh *et al.* [182] proposed extending the native sequence of T20 by two residues on the N‑terminus four residues on the C-terminus. The new peptide, denoted T20^42^ had substantially increased helical propensity over the original peptide, which is a much sought-after method of designing more efficacious fusion inhibitors.

## 5. Future Directions and Concluding Remarks

Overall, targeting mechanistic aspects involving viral fusion mediated by the fusion protein gp41 is a promising approach to develop new anti-HIV therapeutics. However, currently available peptide inhibitors, although effective in the short term, are ultimately unsustainable due to high cost, difficulty of delivery, and susceptibility to mutant forms of HIV. In the absence of a vaccine, a small-molecule fusion inhibitor is urgently needed. Thus far, the deep pocket on the surface of the NHR trimer has been the focal point of much of the small-molecule development. It is highly conserved and it is a proven mode of fusion disruption. However, the community must not neglect other potential avenues of blocking fusion. Other highly conserved motifs or pockets, preferably with complementary inhibition mechanisms to the deep pocket, should be exploited.

A critical step in achieving this ambitious goal is improved understanding of gp41 structural biology including the conformational changes that take place during fusion and its interactions with the membrane and other proteins including gp120. The majority of the gp41 structural data that is currently available is for the pre-hairpin conformation, the post-fusion state, or ectodomain fragments. Interpolation of pathways to intermediate states would be invaluable to the discovery of new modes of inhibition, including for example NHR trimer bundle disruption or disruption of the fusogenic membrane-interacting regions. As outlined in this review, computer-aided approaches are a powerful way to characterize molecular recognition associated with membrane fusion events at the atomic level. These methods have already contributed significantly to the development of currently approved drugs to treat HIV/AIDS and their continued application will ultimately enable design of improved fusion inhibitors targeting gp41.
